# Supplementary material for: Fresh Compost Tea Application Does Not Change Rhizosphere Soil Bacterial Community Structure, and Has No Effects on Soybean Growth or Yield
Source: Plants (Basel). 2021 Aug 10;10(8):1638. doi: 10.3390/plants10081638 (PMC8399032; doi:10.3390/plants10081638)
Supplement: Supplementary file 1 [file plants-10-01638-s001.zip › plants-1302135-supplementary.pdf]

Supplementary Figures

Fresh compost tea application does not change rhizosphere soil bacterial community structure, and has no effects on soybean growth or yield

Bali R.[1], Pineault J.[2], Chagnon P.L.[1], and Hijri M.[1,3]

1. Université de Montréal, Institut de Recherche en Biologie Végétale, 4101 Sherbrooke Est Montréal (QC) H1X 2B2

2. E´comestible Inc, 470 rue Constable McMasterVille, (QC) J3G 1N6, Canada

3. African Genome Center, Mohammed VI Polytechnic University (UM6P), Lot 660, Hay Moulay Rachid, Ben Guerir 43150, Morocco

Supplementary Figures

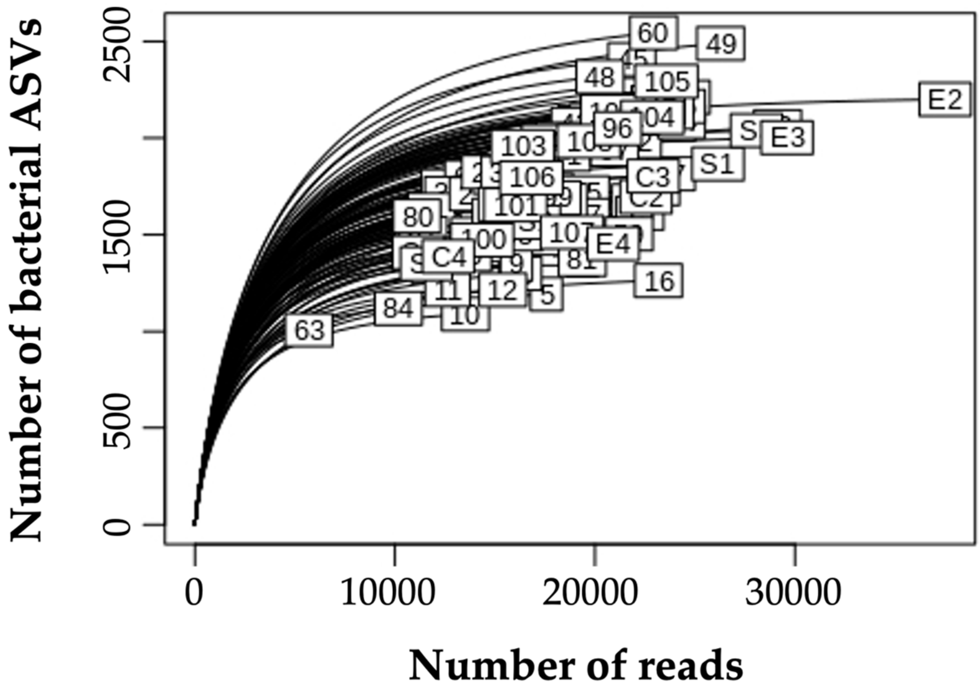

Figure S1. Rarefaction curves of ASVs for individual samples across the different samples.

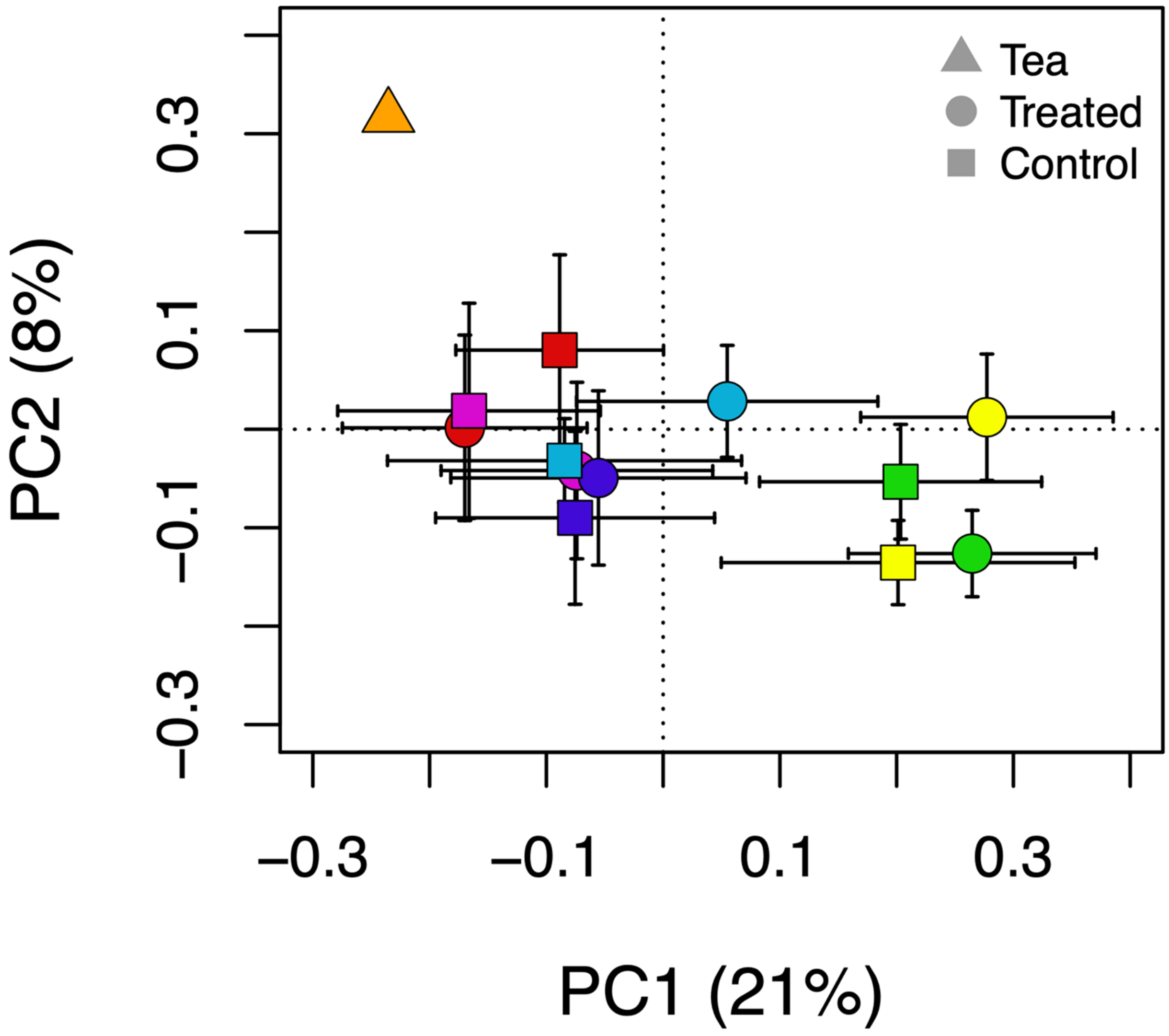

Figure S2. Principal component analysis of Hellinger-transformed relative abundances of Planctobacteria which was the dominant phylum in our dataset. Bacterial communities tend to cluster according to experimental blocks (yellow, green, dark-blue, light-blue, pink and red, represent six experimental blocks; orange color, represents compost tea). Shapes represent treatments.

Supplementary Figures

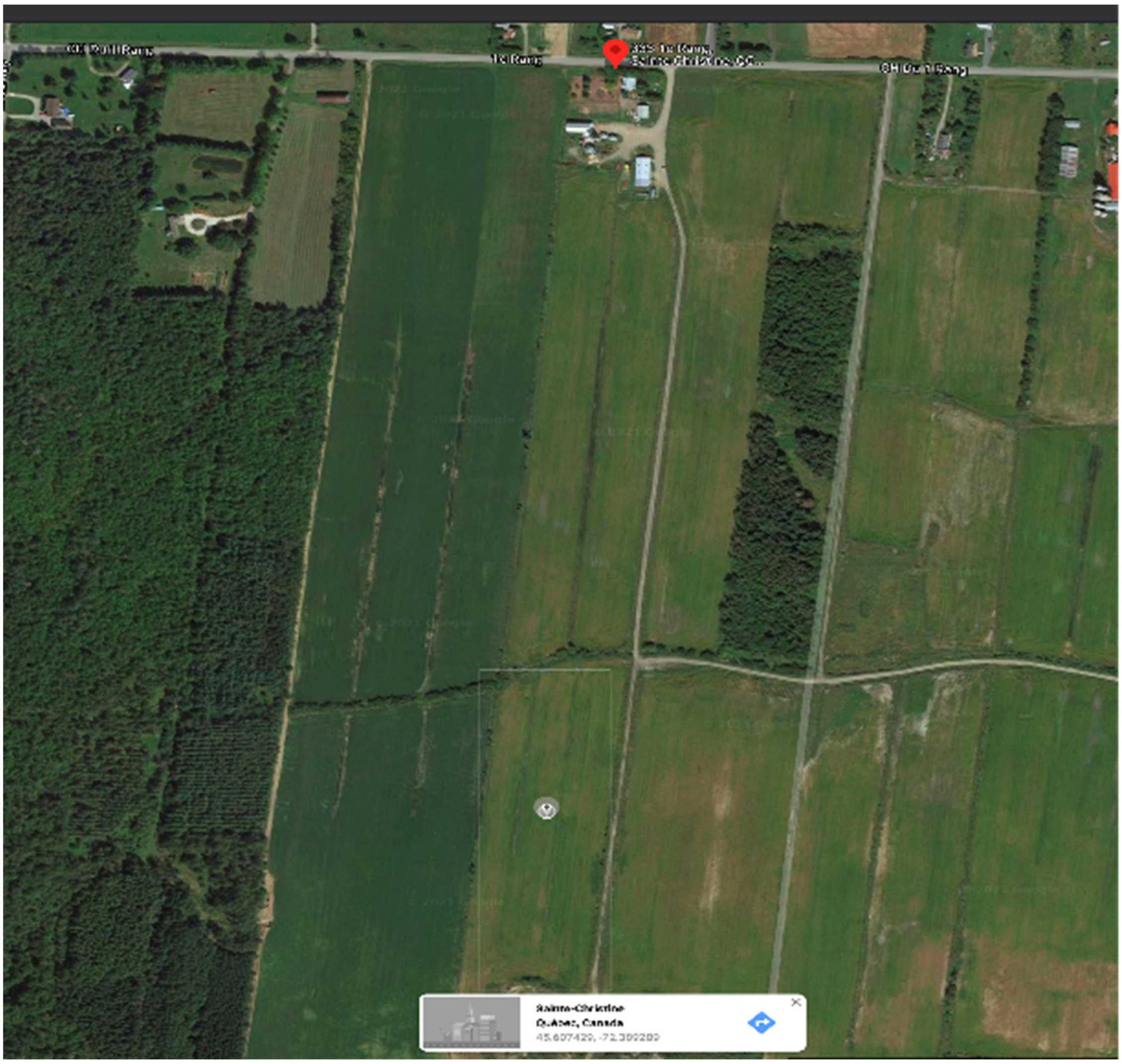

Figure S3. Aerial view of the site, located in Ste-Christine, Montérégie (QC, Canada). The region is dominated by intensive cropping systems, predominantly soybean-maize rotations.

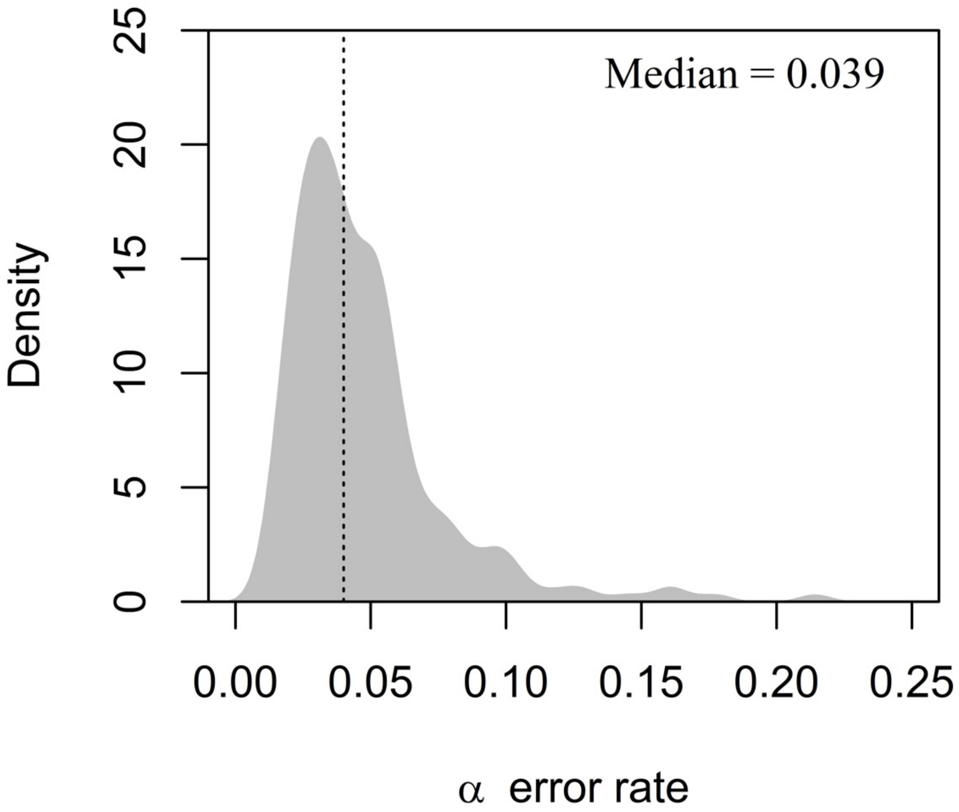

Figure S4. Evaluation of type I error rates in our indicator species analysis. For 1000 random trials, our bacterial metacommunity (i.e., 118 sites [samples] as rows and bacterials ASVs as columns) was randomized using a null model (vaznull in the R package bipartite) that constrains connectance and marginal totals, which is a conservative approach to metacommunity randomization. Then, we ran an indicator species analysis, to evaluate the frequency of false positives that would arise in these trials (i.e., the frequency with which ASVs would be flagged as indicative of a given "treatment" even though the metacommunity had been randomized). For each run, we calculated the proportion of ASVs considered "indicator taxa" as the a-error rate. We report the density plot of this a-error rate for our 1000 random trials. The vertical line report the median value of our 1000 trials, i.e. 3.9%.

Supplementary Figures
